# Addressing shame in medical professional identity: is there such a thing as a good enough doctor?

**DOI:** 10.1192/bjb.2019.70

**Published:** 2019-12

**Authors:** Norella Broderick, Roisin Vaughan

**Affiliations:** Registrar, Cluain Mhuire Services, Blackrock, Co. Dublin. Email: norella.broderick@gmail.com; Senior Registrar, Cluain Mhuire Services, Blackrock, Co. Dublin

The author of ‘Addressing shame in medical professional identity’^1^ is to be congratulated for bringing this topic to the fore of our consciousness. It is welcomed along with the recent surge of popular literature focusing on the emotional challenges of medical practice (particularly among trainees), including Adam Kay's *This is Going To Hurt* and Danielle Ofri's *What Doctors Feel*.

We know that shame affects the self-care of doctors; it increases the risk of mental health problems by making us less likely to access support when needed. Mental health is currently a critical issue, especially among junior doctors, who have higher levels of clinically significant mental health problems than the general population;^2,3^ junior trainees are less likely to disclose mental distress,^2^ and the more junior a doctor is, the less likely they are to know how to access support.^3^ Further, failure to disclose mental distress and access help perpetuates stigma. There remains a high rate of presenteeism,^2^ probably mediated by shame.

We also know that shame affects patient care experiences. The author notes that shame leads to reticence among doctors to disclosure errors. This means that teams are less able to learn from the mistakes of members, and the service does not have the opportunity to improve.

The author discusses what is not helpful in addressing shame (mandated reflective writing) but is vague on practical solutions – although there is a citation of Brown, reflecting that self-compassion is the antidote to shame. To our minds, this notion comes from Prof. Paul Gilbert's school of thinking and his team's extensive research on compassion-focused therapy (CFT) to address shame in a variety of clinical settings. They found that CFT training (a three-day workshop) for healthcare providers increased self-compassion and reduced self-critical judgement in clinicians.^4^

We suggest that CFT training could be provided to doctors as part of our suite of regular training courses. Hand hygiene training is ubiquitous, and we propose that mental health hygiene training is equally important. This could be accessed through the training colleges or from employers directly, like hand hygiene education.

In considering self-compassion as a profession, we encourage doctors to view themselves as ‘good enough’. Drawing on the work of Winnicott^5^ in finding that ‘good enough mothers’ are what babies need, we suggest that ‘good enough doctors’, rather than perfectionist, shamed doctors, are what patients need.

Shame can only be addressed as above if we try to commit to a culture of disclosure and of self-compassion among doctors. As the author above describes, shame is endemic in medicine. As a step towards openness, and towards addressing shame, we ask readers to consider: can I (let myself) be a good enough doctor?
